# Neurogenic bladder pathophysiology, assessment and management after lumbar diseases

**DOI:** 10.1530/EOR-24-0087

**Published:** 2025-03-03

**Authors:** Jiayu Hao, Jian Jiang, Qiaoyue Han, Kui Wang, Yuefeng Sun, Hong Wang

**Affiliations:** ^1^ Dalian Medical University, Dalian, China; ^2^ Department of Spine Surgery, Dalian Municipal Central Hospital, Dalian University of Technology, Dalian, China

**Keywords:** lumbar disease, lumbar disk herniation, cauda equina syndrome, neurogenic bladder, urinary incontinence

## Abstract

- Neurogenic bladder (NB) is a group of bladder and/or urethral dysfunctions caused by neurological lesions, commonly seen in patients with lumbar spine diseases, manifesting as urinary storage and voiding dysfunction, significantly affecting patients’ quality of life.
- Degenerative changes or trauma to the lumbar spine can lead to narrowing of the dural sac, compressing the sacral nerve roots, cauda equina or blood vessels, causing bladder dysfunction and leading to NB.
- Diagnostic methods for NB include history taking, physical examination and noninvasive and invasive tests, such as urodynamic testing and cystoscopy.
- The treatment goals for NB are to protect upper urinary tract function, restore or partially restore lower urinary tract function, improve urinary control, reduce residual urine volume, prevent urinary tract infections and improve patients’ quality of life.
- Treatment methods include conservative treatment, pharmacological treatment, catheterization, neuromodulation and surgical treatment, which should be sequentially administered based on the patient’s specific condition.

## Introduction

With the progression of social aging, the incidence rate of degenerative spinal diseases is increasing year by year (1). Spinal diseases can be accompanied by nerve stimulation and compression of the corresponding segment of the lesion, leading to various neurological symptoms, such as radiating pain, decreased sensation and decreased motor ability (2). Degenerative changes or trauma to the lumbar spine and intervertebral disks may lead to narrowing of the dural sac, which in turn interferes with the sacral nerve roots, cauda equina or blood vessels, causing bladder dysfunction. Neurogenic bladder (NB) is a general term for a group of disorders in which neurological lesions cause bladder and/or urethral dysfunction (i.e. urinary storage and/or voiding dysfunction), leading to lower urinary tract (LUT) symptoms and complications. The severity of NB dysfunction depends on the location, nature and degree of lumbar lesions (3).

**Figure 1:**
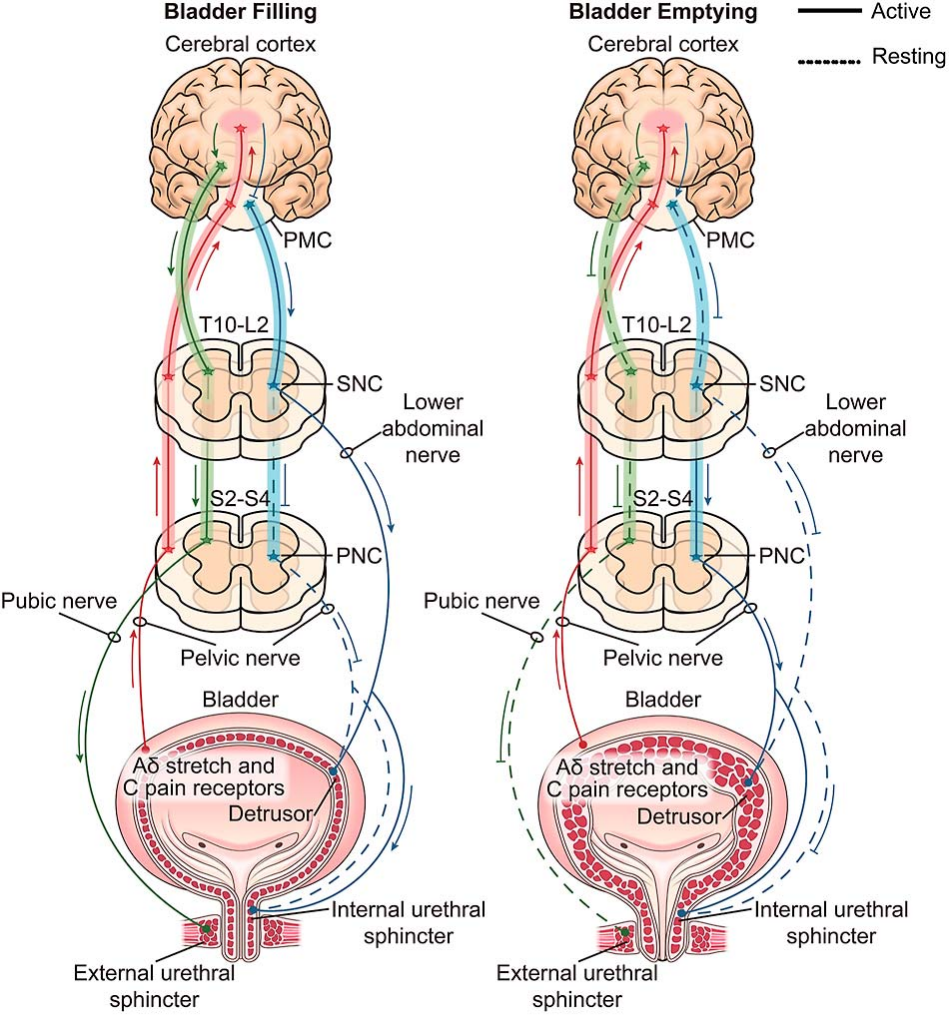
Physiological functional innervation of the LUT. PMC, pontine micturition center; SNC, sympathetic nerve center; PNC, parasympathetic nerve center; LUT, lower urinary tract.

NB severely interferes with patients’ physical and mental health and the health-related quality of life (4). Some studies have found that the proper management of NB is more important for patients than the restoration of walking function (5). Among all the symptoms of NB, urinary incontinence has the greatest impact on the quality of life of patients, which is usually caused by excessive activity of the bladder detrusor muscle, dysfunction of the urethral sphincter muscle or a combination of both (6). Figure 1 shows eight different types of detrusor urethral sphincter dysfunction. Medical intervention may not necessarily restore normal urinary function in patients, but it can improve their quality of life. The diagnostic methods for NB include history, physical examination, noninvasive methods and invasive methods such as urodynamic testing and cystoscopy. Treatment of NB includes multiple nonsurgical and surgical treatment options.

At present, there is little review on the association between NB and lumbar spine diseases, and most discussions are focused on NB itself. This article reviews past literature to identify the latest pathophysiology, diagnosis and treatment regarding lumbar spine disorders causing NB, providing a reference for the clinical treatment of NB caused by lumbar spine disease.

## Pathophysiology

### The innervation of the bladder

The primary function of the bladder is to store and empty urine, and the frequency of voiding in individuals with a bladder capacity of 400–600 mL is every 3–4 h, with the bladder in the storage phase more than 99% of the time (7). The decision to urinate or not is a subjective one, and the factors influencing this decision include the perception of bladder fullness and the acceptance of urination by the surrounding environment (8). The achievement of the voiding function requires communication and coordination between the central nervous system and the peripheral nervous system.

The spinal cord is the primary voiding center that controls the functional activity of the detrusor, the internal urethral sphincters (IUS) and the external urethral sphincters (EUS) and is responsible for transmitting information between the bladder, urethra and the pontine micturition center (PMC). The PMC is located at the pons and controls the contraction of the detrusor and the release of the IUS through the spinal cord. When the PMC is excited, the voiding reflex is triggered. However, tonic inhibition of the PMC by the cerebral frontal cortex maintains bladder filling until the brain deems it appropriate to perform voiding (9). The primary voiding center of the spinal cord contains three main components, namely the sympathetic nerve center (SNC), the parasympathetic nerve center (PNC) and the nucleus accumbens, which send out nerve fibers to innervate the bladder and urethra, respectively. SNC innervates the inferior ventral neurostemming from T10 to L2, which mediates bladder filling by activating α1-adrenergic receptors in the bladder neck and β3-adrenergic receptors at the base of the bladder, causing relaxation of the detrusor. The innervation of bladder activity by the PNC is accomplished via the pelvic splanchnic nerves arising from S2 to S4, causing bladder voiding mainly by its effect on M3 cholinergic receptors in the detrusor. The pubic nerves also arise by S2–S4 and innervate EUS regulated by the autonomic, somatic nerves (9). Nerve damage in the pubic area can lead to reduced bladder capacity and urinary frequency. EUS can prevent bladder emptying, and when EUS contracts involuntarily and simultaneously with the detrusor, it may lead to detrusor sphincter dysfunction, but this is uncommon in the NB caused by lumbar spine disease. Information from the tone receptors and nociceptive receptors of the bladder wall reaches the sacral section of the spinal cord via PNC, which has the most important role in initiating voiding.

### NB caused by lumbar spine disease

The cauda equina nerve is a collective term for ten pairs of lumbosacral nerve roots under the spinal cord cone, which innervates the autonomous control of the urethral sphincter (10, 11). In adults, the lumbosacral nerve roots below the second lumbar vertebra walk vertically downward, passing through the corresponding intervertebral spaces in sequence. Due to the absence of a spinal cord during their journey, they resemble the tail of a horse, hence the name cauda equina (12, 13). Pathological features of lumbar spine disease include enlarged ligaments, herniated disks, osteophytes and narrowing of the lumbar spinal canal and such alterations could compress the cauda equina, nerve roots and blood vessels, interfere with the nerves to the bladder, fundus of the perineum and corpus cavernous tissue of the penis. When a NB occurs, the surrounding nerves and bladder undergo morphological changes. The volume and duration of disk prolapse are associated with these alterations, but not with bladder function recovery (14). Early compression can stimulate the nerve roots and induce the excessive activity of the detrusor, and the associated bladder deformation may manifest as detrusor hypertrophy. As the disease progresses, chronic disk or lumbar spondylosis leads to long-term damage to the nerve roots, nerve compression leading to nutritive damage and nerve demyelination and a gradual decrease in bladder sensitivity. The result is the gradual shrinkage of sensory nerves, detrusors and autonomic and somatic nerves. Eventually, it causes thinning of the detrusor, bladder dilatation, diminished bladder sensitivity and hypoactivity of the detrusor. At this point, the inferior motoneurons are impaired along with the micturition reflex pathway, and PMC maintains its integrity, resulting in the forcefulness of the detrusor and relaxation of EUS, loss of bladder filling sensation and eventually overflow incontinence (15). Cauda equina syndrome (CES) causes LUT dysfunction in four stages. It initially presents with bilateral radicular sciatica and gradually develops urinary symptoms as the disease progresses, such as a change in urination sensation, lack of micturition urge, inappropriate uroflow, the requirement for forceful urination and then painless urinary retention, overflow incontinence and eventually bladder paralysis (16).

## Diagnosis

### Bedside assessment: history, voiding diary and physical examination

During the initial evaluation, all patients must receive a detailed history, voiding diary and physical examination assessment. During history taking, the following aspects ought to be taken into consideration: (A) The pathogenesis, diagnosis, treatment and prognosis of lumbar spine disorders are important in the management of NB. Typical symptoms of CES show that the primary reason for pathogenesis is spinal canal compression (17). (B) Urinary retention due to lumbar spine disease usually precedes incontinence; therefore, in the early stages of the disease, clinicians should not ask about incontinence, but rather about urinary retention and voiding difficulties, and should exclude interference from other urological conditions. (C) Special consideration should be given to factors in a woman’s medical history that may lead to urinary tract infections (UTIs). Female patients are at greater risk of developing UTIs, and it is more difficult to perform intermittent catheterization from the external urethra. (D) Neurological, vascular, skeletal and metabolic disorders may impact bladder evaluation and treatment options (9). A history of serious high blood pressure or acutely closed-angle glaucoma could prohibit the utilization of drugs to manage NB (18). Benign prostatic hypertrophy, urinary frequency and any urological malignancy may produce symptoms similar to NB. (E) Drug hypersensitivity and usage of opioids, anticholinergics, alpha-adrenergic drugs and psychotropic medications ought to be recorded as they might interfere with treatment.

Using a micturition diary, in conjunction with the relevant medical history, onset of the disease, relevant complaints, achiness and chronic conditions, might assist in etiology and a baseline for treatment effectiveness (9). A 3-day micturition diary should include the method of emptying the bladder, the number of voluntary voiding/catheterization sessions, foods high in water content, intake of amount of fluid, duration of voiding, the volume of urination, sensation of urgency and incontinence episodes (4, 19).

The physical examination is performed by a clinician and includes an assessment of lumbar spine disease, abdomen, pelvic, musculoskeletal and derma. Physical examination of patients with CES should concentrate on checking strength and feeling in inferior limbs, perianal sensation and related reflexes, especially the bulbocavernosus reflex. Meanwhile, sensory examination of proprioception, touch, pinprick and reflexes should be performed to understand the function of the nerves in the pubic area. Genital examinations assess for derma lesions and lesions or masses. Physical examination should cover the evaluation of voluntary contractions of the muscles of the perineal region and anal sphincter tone (15). Their presence will help to differentiate between upper motor neuron and lower motor neuron bladders. In addition, the ability of the anal sphincter to contract autonomously may be indicative of bladder conditions during recovery (5). Determine the degree of hand function to consider management methods (self-catheterization, indwelling catheter, nursing staff assistance, toilet, etc.). Cognitive and neurological examinations should be included, as urination includes both voluntary and involuntary. Patients who cannot be checked because of severe pain should have their pain controlled first.

### Auxiliary examinations

#### Noninvasive tests

The gold standard for CES is total or almost total occlusion of the spinal canal as observed on MRI (20). For those patients who can voluntarily void, a post-void residual (PVR) should be obtained, as an increased PVR may suggest a lack of detrusor activity, bladder outlet obstruction or both (19). For those patients who cannot voluntarily void, the pad test is a noninvasive and affordable method of identifying and measuring incontinence by measuring the weight change of a pad or diaper over 24 h. Urine flow rate measurement combines the function of the detrusor and urethral sphincter during urination (19, 21); however, it is only valuable in individuals who urinate spontaneously and have a bladder capacity greater than 150 mL (4). Urinalysis and renal function assessment can exclude UTIs, kidney disease and diabetes. Urine culture can be utilized to determine UTI in patients with symptoms of UTI and other situations (e.g., before performing cytometry). Drug sensitivity test results can be used to guide antibiotic prescription, but antibiotics should not be routinely used in patients with asymptomatic bacteriuria (4, 21).

Renal and bladder ultrasonography is used to investigate the renal size and occupying lesions, cortical thinning, hydronephrosis and urolithiasis, and to determine PVR volume or bladder capacity, diverticula and wall thickening (4). If the ultrasound shows ureteral or renal effusion without underlying superior urinary circuit dysfunction, it indicates an unacceptably high storage pressure in the bladder, a condition that may result in upper urinary tract (UUT) injury, which can involve hydronephrosis, pyelonephritis and kidney failure (22, 23). Hence, it is crucial to identify these processes early and kidney ultrasound is an essential selection instrument (21, 24).

For the evaluation of patients with NB, several neurophysiological methods have been used. Among them, electromyography for the hypodermic external anal sphincter is standardized and can be readily applied. The potential of the public somatosensory triggered signal and sphincter motor-triggered signal might be limited to revealing additional asymptomatic pathological changes and assessing the correlation of these lesions with LUT dysfunction. In an experiment based on an animal model of mouse spinal cord injury, the reduction time of external urinary sphincter-electromyography (EUS-EMG) increased continuously with the duration of the disease (25). In Jiang *et al.*’s study, changes in denervation, reinnervation and reduced EUS recruitment (both reduced and absent) were seen in 21.7, 71.7 and 88.7% of human patients, separately (26). This suggests that EUS-EMG may have diagnostic value for NB due to lumbar spine disease but is limited by the small size of the EUS, which makes it difficult to examine. Among urodynamic examinations, the combined use of pelvic fundus EMG and cystourethrography is the widest accepted approach to diagnose the external sphincter of the detrusor urethra dysfunction (27).

#### Invasive tests

Urinary cystoscopy should be used to evaluate the LUT in patients with anuria combined with hematuria, NB with concomitant hematuria, recurrent uremia, suspected anatomical abnormalities or stones (21). Some studies have shown that cystoscopy and cytology are not effective in screening for bladder cancer in patients with NB, and patients without these signs and symptoms should not be routinely screened (21). Urodynamic studies allow the assessment of the function of the LUT, including the evaluation of detrusor compliance and voiding pressure (15, 21). This is essential to diagnose whether a patient is in a condition that predisposes to upper tract injuries, such as poor bladder compliance, detrusor sphincter dyssynergia (DSD) and bladder outlet obstruction. Clinicians must monitor hemodynamics in individuals at risk for autonomic dysreflexia (AD) throughout the urodynamic study and should terminate the study and empty the bladder if AD occurs. If this approach does not improve hemodynamics, pharmacological treatment should be considered (21). Bladder volume and pressure for which AD occurs ought to be recorded and taken into account by the physician when providing treatment recommendations. Table 1 shows diagnostic of NB due to lumbar spine disorders.

**Table 1:** Diagnostic of NB due to lumbar spine disorders.

Diagnostic method	Content
Medical history	Detailed medical history, including the pathogenesis, diagnosis, treatment and prognosis of lumbar spine disorders
Voiding diary	Use a 3-day voiding diary, including the method of emptying the bladder, the number of voluntary voiding/catheterization sessions, intake of high-water-content foods, fluid intake, duration of voiding, volume of urination, sensation of urgency and incontinence episodes
Physical examination	Examination includes assessment of lumbar spine diseases, abdomen, pelvic, musculoskeletal and skin
MRI	The gold standard is the total or almost total occlusion of the spinal canal as observed on MRI
PVR measurement	For patients who can voluntarily void, measure the PVR volume
Pad test	For patients who cannot voluntarily void, measure the weight change of a pad or diaper over 24 h to identify and measure incontinence
Urine flow rate measurement	Valuable only in individuals who urinate spontaneously and have a bladder capacity greater than 150 mL
Urinalysis and RFA	To exclude UTI, kidney disease and diabetes
Urine culture	Used for patients with UTI symptoms; drug sensitivity test results guide antibiotic prescription
Renal and bladder ultrasonography	Used to investigate renal size, occupying lesions, cortical thinning, hydronephrosis and urolithiasis, and to determine PVR volume or bladder capacity, diverticula and wall thickening
Electrophysiological examinations	EMG of the external anal sphincter is standardized and can be used to evaluate NB
Cystoscopy	Used to evaluate the LUT in patients with anuria combined with hematuria, NB with concomitant hematuria, recurrent uremia, suspected anatomical abnormalities or stones
Urodynamic studies	Assess the function of the LUT, including detrusor compliance and voiding pressure

EMG, electromyography; PVR, post-void residual; RFA, renal function assessment; UTIs, urinary tract infections; NB, neurogenic bladder; LUT, lower urinary tract.

## Treatment

If LUT dysfunction has not improved with treatment of the primary lumbar spine disease, further treatment is required. Treatment goals for NB include primary and secondary goals. The primary goal is to preserve UUT function (protection of renal function) and to ensure that bladder pressure is within safe limits during the storage and voiding periods. The secondary goals are to restore/partially restore LUT function, improve urinary control, reduce residual urine volume, prevent UTIs and improve the quality of patient survival. The choice of treatment should follow a gradual progression from noninvasive, minimally invasive and then invasive. The current treatment of the NB includes conservative treatment, pharmacological treatment, catheterization, neuromodulation and electrical nerve stimulation and surgical treatment.

### Conservation treatment

#### Bladder reflex trigger

The bladder reflex trigger method is used to induce bladder contraction by activating Aδ stretch and C-pain receptors via knocking on the patient’s abdominal area two times each per second (28). However, this method causes the external urethral sphincter to contract involuntarily at the same time, leading to dysregulation of the detrusor sphincter and increasing bladder pressure beyond safe limits and is therefore not recommended (9, 28). If this approach has to be used, the functional status of LUT must be clarified by urodynamics before implementation.

#### Behavioral training

Behavioral training consists mainly of timed voiding and prompted voiding. Timed voiding refers to voiding at defined time intervals and is mainly used in patients who can urinate spontaneously, and it is effective in reducing incontinence due to involuntary detrusor contractions (29). Prompted voiding refers to educating patients to be able to ask for assistance when they want to urinate, requiring the assistance of a third party to do so. This method is suitable for patients with good cognitive function and a sense of bladder fullness, but who are highly dependent on the assistance of others. Modifying drinking habits by spreading water intake over the day, and limiting fluid intake in some cases, can similarly reduce incontinence and prolong catheterization intervals (29). Such management protocols need to be carefully individualized for each patient, with confirmation that the patient has both neurogenic bowel and chronic constipation to avoid exacerbating symptoms due to inadequate fluid intake. Penis sleeves and external urinary collectors can be taken into consideration for males with incontinence, but wearing an external collector can be difficult in patients who are overly obese and have an atrophied or retracted penis. To prevent latex allergies, silicone external urinary collectors with self-adhesive properties are recommended.

### Drug therapy

Pharmacological treatment of NB usually targets the neuromuscular connections of the detrusor or the detrusor of IUS. The goal of treatment is to minimize the episodes of incontinence due to overactivity of the detrusors or to reduce detrusor pressure in the urinary reserve period so that the threat of UUT complications is reduced.

#### Anticholinergic drugs

Anticholinergic agents’ inhibition of the association of M2 and M3 receptors to acetylcholine in the detrusor causes relaxation of the detrusor. This improves symptoms such as incontinence, urgency and frequency, but also decreases the contraction of the detrusors leading to increased residual urine volume; thus, some patients require the addition of intermittent catheterization. Frequently utilized anticholinergic medicines include darifenacin, fesoterodine, oxybutynin, propiverine, solifenacin, tolterodine and trospium chloride (8, 19). These anticholinergic drugs differ from each other only in their side effects and tolerance profiles; thus, if one drug is ineffective or has too many side effects, another drug of this class can still be tried (8, 30).

#### β3-adrenergic receptor agonist

Mirabegron is a β3-adrenergic receptor agonist, which has been utilized in the treatment of overactive bladder. Activation of β3-adrenergic receptors leads to a relaxed detrusor and a decrease in pressure, thereby improving bladder storage. Compared to anticholinergic drugs, this drug does a better job of increasing bladder capacity, reducing bladder end-filling pressure, improving bladder compliance, reducing incontinence and reducing cognitive adverse effects (9, 31, 32, 33, 34). Combination therapy using mirabegron with α-receptor antagonists has also been demonstrated to be more beneficial for cases with an overactive bladder, which cannot be controlled with alpha-blockers alone (35). Notably, however, some studies have concluded that although mirabegron improves maximal bladder capacity, compliance and maximal detrusor pressure, these improvements are not statistically significant (31, 34, 36). Vibegron, a newer β3-adrenoceptor agonist, has also been demonstrated as useful for the control of overactive bladder individually or in association with antiarrhythmic agents, for example, tolterodine (37).

#### α-receptor inhibitor

α-receptor inhibitors relax the bladder neck and reduce efflux resistance. Medicines, for example, tamsulosin and terazosin reduce incontinence, improve bladder volume and emptying and diminish the rate of AD, especially in men (9, 19). Adverse effects involve postural low blood pressure, dizziness and erectile disorder with tamsulosin (28).

#### Intravesical administration

For patients who cannot tolerate the side effects of oral drugs, intravesical administration is a good method, such as intravesical instillation of oxybutynin, the fiber-sensitive neurotoxin, capsaicin and its analogs, the local anesthetic bupivacaine hydrochloride and C-nerve, all of which have achieved some efficacy. Intravesical infusion of anticholinergic drugs inhibits the hyperreflexia of the detrusor while effectively reducing the systemic side effects of anticholinergic drugs. Capsaicin and resiniferatoxin (RTX), both C-fiber blockers, reduce the overactivity of the detrusor by desensitizing the C-fiber, and their effects are maintained until the C-fiber resensitizes. In addition, drugs such as desmopressin, potassium channel opener and neurokinin receptor antagonist have certain therapeutic effects on the NB.

### Urethral catheterization treatment

Retaining catheterization for too long or ending it at the wrong time could raise risks of UTIs and postoperative infections and consequently affect the patient’s life quality (5). Currently, clean intermittent catheterization is the routine to manage inadequate voiding. It does not require a prolonged indwelling catheter and permits a more natural filling and emptying of the bladder (19, 38). In contrast, indwelling catheters and suprapubic catheters stay inside the urethra longer, increasing the risk of LUT infection. However, in female patients, intermittent catheterization from the external urethral opening is difficult; thus, indwelling catheters or pads are commonly used. Notably, long-term indwelling urinary catheters or cystostomies are associated with more complications; thus, such patients are followed-up at a minimum of once a year including urodynamic testing, kidney function tests and total urinary tract imaging. External condom catheters are available for use in male patients who are incontinent or using reflex urination methods, but compression of the penis from the device may lead to lesions and ischemia, especially in cases of dementia and hyposensory limbs (30).

### Surgical treatment

When planning surgical treatment, physicians need to consider the patient’s LUT symptoms, impairment degree and test results. Common surgical treatments for NB can be divided into procedures that expand bladder capacity, procedures that increase urethral urinary control and modalities that address voiding disorders. For NB caused by lumbar spine disease, unless medically contraindicated, CES is treated by decompression of the cauda equina nerve. The study by Sangondimath *et al.* showed that after CES surgery, age at onset and gender do not affect the recovery of bladder function, but are directly correlated with the number of days surgery is delayed and the presence of unilateral leg pain (39).

#### Procedure to expand bladder capacity

##### Injection therapy

Botulinum toxin type A bladder wall injections are preferred in patients who have failed conservative treatment but whose bladder wall has not yet become fibrotic. Botulinum toxin interrupts the synaptic activity at the nerve–muscle junction by reducing the release of acetylcholine (40, 41, 42). Regardless of the etiology, intramuscular botulinum toxin injections in the detrusor are a safe and useful way to reduce symptoms of neurogenic detrusor hyperactivity (19, 40, 43, 44). Targeted engineering of new botulinum toxoids has better results (45). Bladder augmentation is preferred in patients in whom botulinum toxin injections are ineffective or in whom repeated injections are not available and in patients who have developed severe bladder wall fibrosis, bladder contracture and combined severe vesicoureteral reflux.

##### Bladder dilation

Bladder dilation is indicated in patients with high bladder pressure and low detrusor compliance who have failed to respond to oral therapy and bladder chemoprotection (46). It has the advantage of allowing access to the UUT through the native ureteral orifice, thus maintaining the integrity of the native urinary tract. Any bowel segment can be used to enlarge the bladder and their improved compliance, incontinence and life quality are consistent (47). In clinical practice, the most frequent choices are ileal and colonic. The most significant absolute contraindication to bladder expansion would be the impossibility of intermittent catheterization, as in the case of tetraplegia, or in patients who do not want to have interval catheterization. Bladder dilation should not be an option for cases with histories of bladder carcinoma. Some potential complications of this procedure include the occurrence of adenocarcinoma or urothelial cancer, anastomotic leakage, infection, UTI, bowel obstruction, bladder stone formation and bladder rupture (29, 46).

#### Modalities to manage voiding disorders

##### Neuromodulation

Neuromodulation techniques were initially prohibited for the treatment of NB and can only be used for non-NB overactivity (48). Several small-scale heterogeneity trials have extrapolated the use of this technology to the population with NB (49, 50). Neuromodulation blocks competitive afferent signals from the bladder by stimulating the peripheral afferent nerves and prevents reflex overactivity or retention of the bladder (51, 52, 53).

Sacral nerve modulation (SNM) has become a common treatment method for patients with refractory LUT dysfunction (54, 55, 56, 57, 58, 59). SNM surgery is performed in two phases. In the first stage of surgery, insert the electrode wire into the S3 foremen, place it and connect the electrode wire to an external pulse generator. Subsequently, the patient will be observed for two weeks. If the patient’s symptoms improve by 50% or more, the electrode wire will be connected to the implantable pulse generator and fixed to the upper part of the skull during the second phase of surgery. Otherwise, the electrode wire will be removed (49, 60). In the study by Weil *et al.*, SNM was significantly more effective than conservative treatment (61). In the study by Amundsen *et al.*, the efficacy of injecting botulinum toxin type A was superior to that of SNM (62). Although studies have shown that NB patients can benefit from SNM, further discussion is needed on whether it can be considered as one of the treatment options for NB patients.

Pudendal neuromodulation (PNM) is also one of the possible treatment options for NB patients. In the study by Chen *et al.*, PNM can increase bladder capacity in the early stages of spinal cord injury (63). However, further discussion is needed on whether PNM can be applied in the early stages of spinal cord injury and its impact on the pathological changes of the bladder.

In addition, there are methods such as pelvic nerve electrostimulation, pelvic floor muscle electrical stimulation, foot stimulation and direct electrical stimulation of the detrusors.

##### External urethral sphincterotomy

For male patients available and consenting to use a condom catheter, an external urethral sphincterotomy and/or an internal urethral stent could be beneficial to aid in the complete emptying of a high-pressure bladder. Unfortunately, the failure rate of external urethral sphincterotomy is high, with 78% of cases having to perform as many as three repeat sphincterotomies (19).

##### Continuous urinary diversion (CUD)

CUD surgery permits fluid gathering within the body, demands the capability to drain urine at intervals by catheterization or voiding and is an incontinence technique of choice (46). CUD includes both types of self-contained skin reservoirs and bladder substitution (64). Incontinent urinary diversion (IUD) is usually considered a last resort option (29). IUD creates a pathway that continuously diverts urine to an intra-abdominal pouch connected to the stoma, which is indicated for patients with impaired renal function and inability to perform catheterization (65). Several types of IUD can be performed, including ileal catheterization, cutaneous ureterostomy, ileostomy and ureterostomy. The treatment methods for NB caused by lumbar spine diseases are shown in Table 2.

**Table 2:** Treatment of NB due to lumbar spine diseases.

Category	Method
Conservation treatment	Bladder reflex trigger, behavioral training, penis sleeves and external urinary collectors, and modifying drinking habits
Drug therapy	
Anticholinergics	Darifenacin, fesoterodine, oxybutynin, propiverine, solifenacin, tolterodine and trospium chloride
β3 adrenergic receptor agonists	Mirabegron and vibegron
Alpha-adrenergic antagonists	Tamsulosin and terazosin
Intracavitary perfusion therapy	Oxybutynin, capsaicin and its analogs and local anesthetic buprocaine hydrochloride
Others	Desmopressin, potassium channel opener and neurokinin receptor antagonist
Urethral catheterization treatment	Clean intermittent catheterization, indwelling urethral catheters, suprapubic catheters and external condom catheters
Surgical treatment	
Procedures to expand bladder capacity	Botulinum toxin bladder wall injection/bladder chemoprotection and bladder dilation
Procedures to increase urethral control	Artificial urethral sphincter implantation, periurethral filler injection, urethral sling and femoral thin muscle urethral myoplasty
Modalities to manage voiding disorders	Neuromodulation, external urethral sphincterotomy and CUD

NB, neurogenic bladder; CUD, continuous urinary diversion.

## Conclusions

Lumbar diseases may interfere with the corresponding spinal cord or nerve roots, leading to NB. The severity of NB depends on the progression of the lumbar spine disease. NB has a negative impact on the quality of life of patients with lumbar spine diseases; therefore, its diagnosis and treatment are crucial for patients. The future diagnosis and treatment of NB are likely to become more precise, personalized and comprehensive. Improved diagnostic technologies, such as machine learning, artificial intelligence and big data predictive models, and more advanced imaging techniques will enhance diagnostic accuracy and efficiency.

## ICMJE Statement of Interest

The authors declare that the research was conducted in the absence of any commercial or financial relationships that could be construed as a potential conflict of interest.

## Funding Statement

This work was supported by the Dalian Medical Science Research Program Project (grant no. 2111005), Guang Zhou Science and Technology Plan Project (grant number 202102020033), the Natural Science Foundation of Liaoning Provincehttps://doi.org/10.13039/501100005047 (grant number 2022-MS-443) and the Dalian University of Technologyhttps://doi.org/10.13039/501100002980 and Affiliated Central Hospital joint research fund (2022ZXYG45).
